# Motivation to Learn at the Intersections of Age, Gender, and Race

**DOI:** 10.1093/geroni/igab046.3159

**Published:** 2021-12-17

**Authors:** Takashi Yamashita, Thomas Smith, Shalini Sahoo, Phyllis Cummins

**Affiliations:** 1 University of Maryland, Baltimore County, Baltimore, Maryland, United States; 2 Northern Illinois University, DeKalb, Illinois, United States; 3 Miami University, Oxford, Ohio, United States

## Abstract

Continuing adult education and training, or lifelong learning, has become increasingly important to fully engage in rapidly changing technology and information-rich societies. However, without motivation to learn (MtL), lifelong learning participation is unlikely to occur. Although previous research has identified lifelong learning gaps by various demographic characteristics, including age, gender, and race/ethnicity, little is known about the intersectionality or differences in MtL across specific sub-groups (e.g., older Black women vs. older Black men) at the national level. The current study analyzed U.S. data from the 2012/2014/2017 Program for International Assessment of Adult Competencies (PIAAC) to examine MtL at the intersections of age (five 10-year age groups), gender (women vs. men), and race (White vs. Black). The previously established 4-item latent MtL construct was evaluated for twenty sub-groups using the alignment optimization method, which is a machine learning algorithm for latent mean estimation and simultaneous multiple group comparisons. Results showed that the latent MtL construct was validly measured across the sub-groups, and the estimated sub-group means were then used to develop a national MtL profile. Overall, older adults tended to have lower MtL than younger age groups. Notably, compared to than older Black men age 66+ years, older White men aged 55-65 and 66+ years old had lower MtL (latent mean differences of -0.29 and -0.41, respectively, p < .05). Additionally, older Black women had significantly lower MtL than older Black men (latent mean difference = -0.50, p < .05). The national MtL profiles, the intersectionality and policy implications were discussed.

